# Regulation of Energy Stores and Feeding by Neuronal and Peripheral CREB Activity in *Drosophila*

**DOI:** 10.1371/journal.pone.0008498

**Published:** 2009-12-30

**Authors:** Koichi Iijima, LiJuan Zhao, Christopher Shenton, Kanae Iijima-Ando

**Affiliations:** 1 Laboratory of Neurodegenerative and Metabolic Diseases, Farber Institute for Neurosciences, Department of Biochemistry and Molecular Biology, Thomas Jefferson University, Philadelphia, Pennsylvania, United States of America; 2 Laboratory of Neurogenetics and Pathobiology, Farber Institute for Neurosciences, Department of Biochemistry and Molecular Biology, Thomas Jefferson University, Philadelphia, Pennsylvania, United States of America; Columbia University, United States of America

## Abstract

The cAMP-responsive transcription factor CREB functions in adipose tissue and liver to regulate glycogen and lipid metabolism in mammals. While *Drosophila* has a homolog of mammalian CREB, *dCREB2*, its role in energy metabolism is not fully understood. Using tissue-specific expression of a dominant-negative form of CREB (DN-CREB), we have examined the effect of blocking CREB activity in neurons and in the fat body, the primary energy storage depot with functions of adipose tissue and the liver in flies, on energy balance, stress resistance and feeding behavior. We found that disruption of CREB function in neurons reduced glycogen and lipid stores and increased sensitivity to starvation. Expression of DN-CREB in the fat body also reduced glycogen levels, while it did not affect starvation sensitivity, presumably due to increased lipid levels in these flies. Interestingly, blocking CREB activity in the fat body increased food intake. These flies did not show a significant change in overall body size, suggesting that disruption of CREB activity in the fat body caused an obese-like phenotype. Using a transgenic CRE-luciferase reporter, we further demonstrated that disruption of the adipokinetic hormone receptor, which is functionally related to mammalian glucagon and β-adrenergic signaling, in the fat body reduced CRE-mediated transcription in flies. This study demonstrates that CREB activity in either neuronal or peripheral tissues regulates energy balance in *Drosophila*, and that the key signaling pathway regulating CREB activity in peripheral tissue is evolutionarily conserved.

## Introduction

Energy balance is maintained by concerted changes in behavior and metabolism, which are often regulated by gene expression [1], [2], [3], [4]. The cAMP responsive element binding protein (CREB) is an evolutionarily conserved transcription factor that is involved in many physiological functions including energy metabolism [4], [5], [6], [7]. In response to pancreatic glucagon and adrenal cortisol, CREB activates gluconeogenic and fatty acid oxidation programs in mammals [1]. Blocking CREB activity in mammalian liver causes excessive fat accumulation and eventually “fatty liver”, which has been ascribed to overactivation of the liposynthesis program [2].

CREB belongs to the activating transcription factor (ATF)/CREB family of proteins. The two major subgroups of the ATF/CREB family are CREB and ATF-2 [8], [9]. ATF-2 activates transcription of the phosphoenolpyruvate carboxykinase-cytosolic (PEPCK-C) gene, which encodes a key enzyme of both gluconeogenesis [10] and glyceroneogenesis [11]. Recently, it has been reported that ATF-2 regulates fat metabolism in *Drosophila*: knock-down of the *Drosophila* homolog of ATF-2 reduced, and overexpression of ATF-2 increased, lipid stores [12].

While *Drosophila* has a homolog of mammalian CREB, *dCREB2* [13], its role in energy metabolism has not been elucidated. The neuronal CREB pathway has been implicated in energy metabolism in flies. The CREB Regulated Transcriptional Coactivator (CRTC, also known as TORC) family of latent cytoplasmic coactivators stimulate CREB-mediated transcription [14], [15]. Flies lacking a homolog of mammalian *TORC* [16], [17] are viable and fertile, but have reduced glycogen and lipid stores and are sensitive to starvation and oxidative stress [4]. Stress sensitivity, reduced energy stores, and CREB target gene expression in *TORC* mutants is rescued by neuronal TORC expression [4], indicating that CREB activity in neurons regulates energy metabolism in flies. In *Drosophila*, the fat body is the primary energy tissue for the storage of fuel molecules, such as glycogen and triglycerides, and adopts the similar functions as mammalian adipose and hepatic tissues. The role of dCREB2 in the fat body in energy metabolism is not clear.

CREB has been found to mediate effects of catecholamines and other fasting hormones on cellular gene expression [1], [18]. For example, the pancreatic hormone glucagon activates the CREB-mediated gluconeogenetic program in the liver in response to a low glucose level during fasting. In insects, the adipokinetic hormone (AKH) pathway is the functional analogue of mammalian glucagon and β-adrenergic signaling [19], [20], [21], [22]. It is unknown whether the AKH pathway regulates CREB activity in *Drosophila*.

Here we demonstrate that energy stores and feeding behavior are controlled by CREB activity either in neurons or in the fat body, and that CREB activity is regulated by the AKH pathway in flies. Interestingly, flies with disruption of CREB functions in the fat body shows an obese-like phenotype.

## Results

### Knock-down of dCREB2 expression in adult flies causes a reduction in glycogen and lipid stores

**Figure 1:**
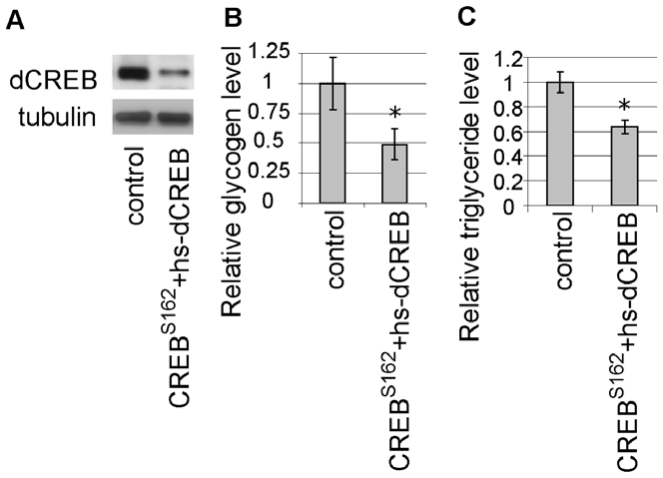
Lower stored glycogen levels in adult flies with loss of dCREB2. (A) A reduction in the CREB level was confirmed by Western blot analysis. A low level of expression of a dCREB transgene (hs-dCREB2d) was used to rescue the lethality of CREB^S162^ (CREB^S162^+hs-dCREB) [23]. Tubulin was used to confirm equal protein loading in each lane. (B) Glycogen content in loss-of-function dCREB mutant flies. Glycogen levels are expressed as ratios to the control level, n = 6, *p<0.05, Student's t-test.). (C) Lipid content in loss-of-function dCREB mutant flies. Lipid levels were normalized to protein levels and expressed as ratios to the normalized control level, n = 4, *p<0.05, Student's t-test.).

To test whether loss of dCREB2 functions causes similar changes in energy stores as the *TORC* mutation, we examined glycogen and lipid levels in flies with a null mutation in *dCREB2*, a *Drosophila* homolog of mammalian CREB/CREM [13]. A loss-of-function mutation in *dCREB2*, *S162*, is lethal [23]. dCREB2 gene has been reported to produce a number of alternatively spliced isoforms (dCREB2a, b, c, d, q, r and s) [24], and the lethality of S162 can be rescued by induction of the dCREB2d isoform under control of the heat shock promoter (hs-dCREB2d) during development [23]. *S162* flies carrying hs-dCREB2d were subjected to a daily 60 min heat shock at 37°C during the embryonic, larval and pupal stages. After eclosion, flies were kept at 18°C for two weeks to shut down hs-dCREB2d expression. This treatment caused a dramatic reduction in dCREB2 levels in adults (Figure 1A).

In *Drosophila*, glycogen and triglyceride comprise the major forms of energy storage for carbohydrate and lipids, respectively. We tested the levels of glycogen and triglyceride in *dCREB2* mutant flies under normal feeding conditions. We found that a mutation in *dCREB2* caused a reduction in stored glycogen levels and a reduction in lipid levels (Figure 1B and C), as was seen in TORC mutants. These results confirm that dCREB2 mediates the regulation of energy stores at the adult stage in *Drosophila*.

### Blocking CREB activity in neurons reduces glycogen and lipid stores

**Figure 2:**
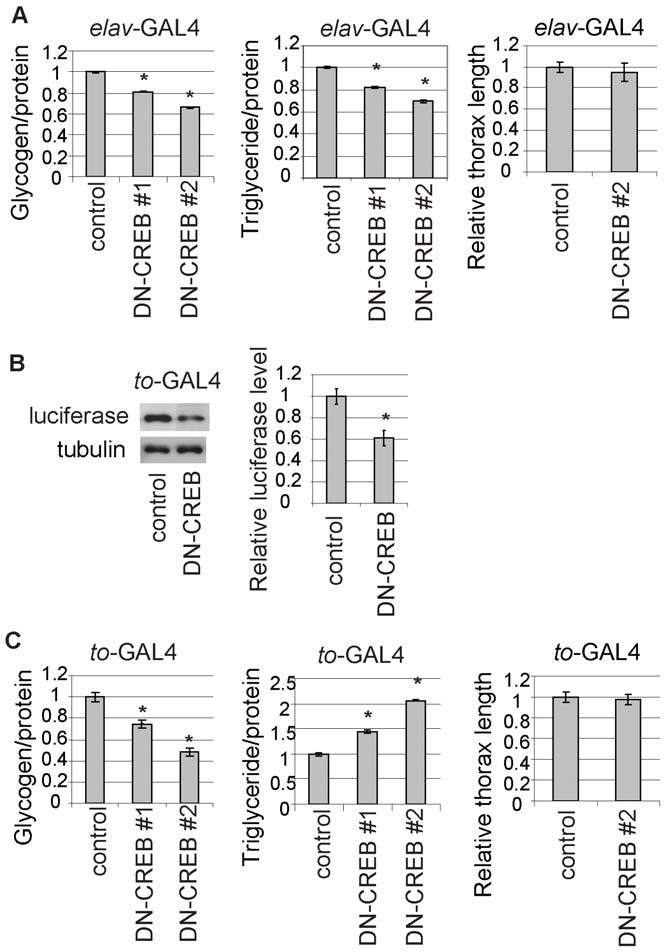
Roles of neuronal and peripheral CREBs in energy stores. (A) Neuronal overexpression of a dominant-negative form of CREB (DN-CREB) caused lower stored glycogen and lipid levels in flies. Glycogen (left) or lipid (middle) content in bodies from control flies (*elav*-GAL4 driver only, control) or flies expressing DN-CREB in neurons from the *elav*-GAL4 (DN-CREB) driver. Glycogen and lipid levels were normalized to protein levels and expressed as ratios to the control level (mean±SD, n = 4, *p<0.05, Student's t-test). Body size (right) of flies with DN-CREB expression in neurons was indistinguishable from that of the control. Measurements of mesothorax size are shown as ratios to the control size (mean±SD, n = 8). Two independent transgenic lines (DN-CREB#1 and DN-CREB#2) gave similar results. (B) Reduction in CREB activity in flies following DN-CREB expression in the fat body. CRE-Luciferase reporter protein was measured using anti-luciferase antibody in Western blots of body extracts from control flies (*to*-GAL4 driver only, control) or flies expressing DN-CREB in the fat body from the *to*-GAL4 driver (DN-CREB) (top panel). Blots were stripped and reprobed with anti-tubulin antibodies as a protein loading control (bottom panel). Signal intensities were quantified and are shown as ratios to control signals (mean±SD, n = 5; *p<0.05, Student's t-test). (C) Overexpression of DN-CREB in the fat body caused lower stored glycogen and higher lipid contents. Glycogen (left) or lipid (middle) content in the bodies of control flies (*to*-GAL4 driver only, control) or flies expressing DN-CREB in neurons from the *elav*-GAL4 (DN-CREB) driver. Glycogen and lipid levels are expressed as ratios to the control levels, n = 6, *p<0.05, Student's t-test). Body size (right) of flies expressing DN-CREB in the fat body was indistinguishable from that of the control. Measurements of mesothorax size are shown as ratios to the control values (mean±SD, n = 8).

Overexpression of an isoform of dCREB2, dCREB2b, has been reported to block CREB activity, presumably by a dominant-negative effect [13], [25], [26], and used to analyze the role of CREB activity in various contexts [25], [27], [28], [29]. To examine whether blocking CREB activity in neurons causes reductions in glycogen and triglyceride levels, we expressed dCREB2b (a dominant negative form of CREB, DN-CREB) in all neurons under the control of the pan-neuronal *elav-*GAL4 driver. We previously reported that CREB activity was reduced in these flies using a transgenic CRE-Luciferase reporter [26]. Similar to the observation in TORC mutant flies [4], we found that neuronal overexpression of DN-CREB caused reductions in stored glycogen and triglyceride (Figure 2A, left and middle). Flies that expressed DN-CREB in neurons did not display reduced body size (Figure 2A, right), indicating that the lower total body glycogen and triglyceride content of these flies is not due to a reduction in overall body size. Similar results were obtained from two independent DN-CREB transgenic fly lines. These results support the previous observation [4] that reduction in CREB activity in neurons causes lower glycogen and lipid stores.

### Blocking CREB activity in the fat body reduced glycogen levels, but increased lipid levels

CREB-mediated transcription mediates glycogen and lipid metabolism in mammalian adipose tissues and liver [1], [2]. The *Drosophila* fat body is the primary energy storage tissue and serves as a repository for both glycogen and triglycerides, thereby combining the energy storage functions of adipose tissues and the liver [30]. To examine whether blocking CREB activity in the fat body alters glycogen and triglyceride levels in flies, we expressed the DN-CREB transgene under the control of the *take out (to)*-GAL4 [31], which drives expression mainly in the fat cells in the head and throughout thorax and abdomen in adult fly bodies [31].

We first confirmed that CREB activity was reduced in these flies using the CRE-Luciferase reporter transgene (Figure 2B). The overexpression of DN-CREB in the fat body caused significant reductions in stored glycogen levels (Figure 2C, left). Interestingly, we found that blocking CREB activity in the fat body significantly increased triglyceride levels (Figure 2C, middle). Flies with expression of DN-CREB in the fat body did not display altered body size (Figure 2C, right), indicating that reduction in glycogen levels is not due to a reduction in overall body size. In addition, the higher total body triglyceride content without an increase in overall body size indicates an obese-like phenotype for flies with DN-CREB expression in the fat body.

### Blocking CREB activity in neurons or in the fat body altered stress resistance in flies

**Figure 3 pone-0008498-g003:**
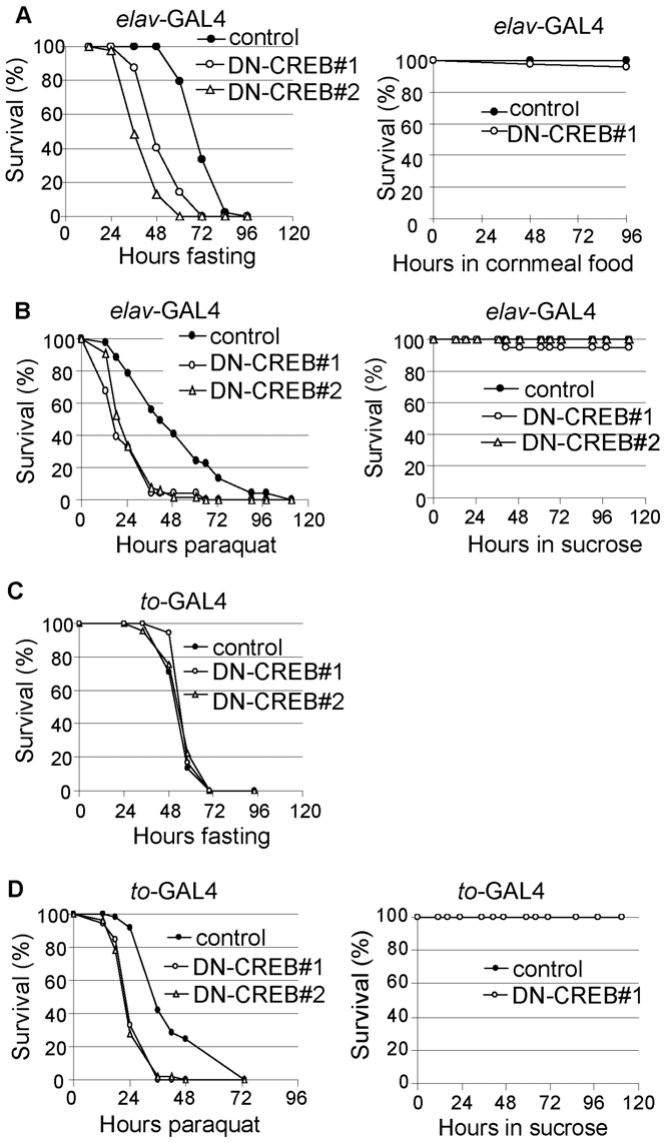
Roles of neuronal and peripheral CREBs in starvation and oxidative stress. (A) DN-CREB overexpression in neurons reduces starvation resistance in flies. (Left) Survival on water only of 7-day-old male flies expressing DN-CREB in neurons (DN-CREB#1 and #2, open circles and open triangles) or control carrying the driver only (filled circles). Two independent transgenic lines (DN-CREB#1 and DN-CREB#2) gave similar results. (Right) Survival on cornmeal food of male flies expressing DN-CREB in neurons (open circles) or control (filled circles). (B) DN-CREB overexpression in neurons reduces oxidative stress-resistance in flies. (Left) Survival in 20 mM paraquat of 7-day-old male flies expressing DN-CREB in neurons (DN-CREB#1 and #2, open circles and open triangles) or control carrying the driver only (filled circles). Two independent transgenic lines (DN-CREB#1 and DN-CREB#2) gave similar results. (Right) Survival on sucrose agar of male flies expressing DN-CREB in neurons (open circles and open triangles) or control flies (filled circles). (C) DN-CREB overexpression in the fat body does not alter starvation resistance in flies. Survival on water only of 7-day-old male flies expressing DN-CREB in the fat body (DN-CREB#1 and #2, open circles and open triangles) or control flies carrying the driver only (filled circles). (D) DN-CREB overexpression in the fat body reduces oxidative stress-resistance. (Left) Survival in 20 mM paraquat of 7-day-old male flies expressing DN-CREB in the fat body (DN-CREB#1 and #2, open circles and open triangles) or control carrying the driver only (filled circles). (Right) Survival on sucrose agar of male flies expressing DN-CREB in the fat body (open circles) or control flies (filled circles).

We found that neuronal expression of DN-CREB sensitized flies to starvation stress (Figure 3A left). In response to water-only starvation, they lived an average of 53 hours, while the control flies lived an average of 74 hours (Figure 3A left). These flies were also sensitive to oxidative stress: following exposure to paraquat, their mean survival time was 50% lower than that of controls (Figure 3B left). When supplied with regular cornmeal food or sucrose, the survival of DN-CREB flies at this age (about 7 days after eclosion) was comparable to that of control flies, suggesting that the reduced survival rates of these flies under stressed conditions are not due to a general “sickly” effect (Figure 3A right and 3B right).

We next tested the effect of blocking CREB activity in the fat body on stress resistance. Interestingly, we found that flies expressing DN-CREB in the fat body were as resistant to starvation as control flies (Figure 3C). Previous work in *Drosophila* has shown that starvation resistance correlates strongly with lipid store levels [32]. Although flies with DN-CREB expression in the fat body had lower glycogen levels, these flies had elevated lipid levels (Figure 2C), which may explain why they were as resistant to starvation as the control flies. In contrast, flies with expression of DN-CREB in the fat body were more sensitive to oxidative stress (Figure 3D left), although their survival was comparable to that of control flies in the absence of oxidative stress (Figure 3D right).

### Blocking CREB activity in the fat body increased food intake in flies

**Figure 4 pone-0008498-g004:**
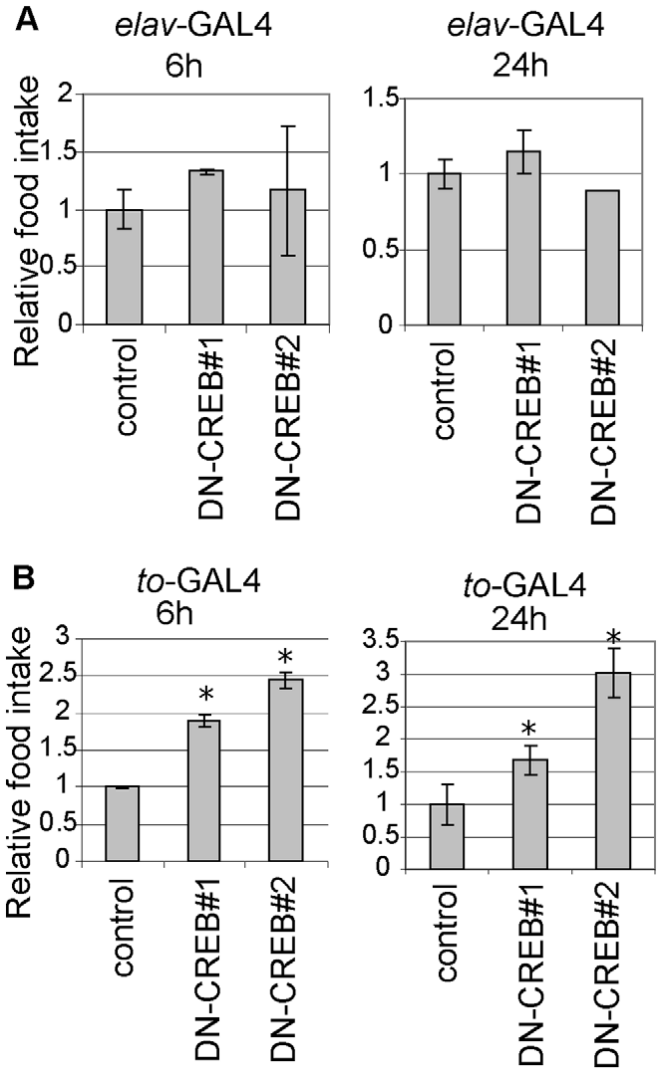
Roles of neuronal and peripheral CREBs in food intake. The ingestion of dye was quantified after feeding 1-week-old male flies for 6 h (left) or 24 h (right). The absorbance of ingested dye was measured and the results are shown as ratios to the control value (mean±SD, *p<0.05, Student's t-test). (A) Flies with DN-CREB expression in neurons. (B) Flies with DN-CREB expression in the fat body.

Changes in food intake behavior can affect energy stores in flies. To test whether DN-CREB expression in neurons or in the fat body alters feeding, we quantified food intake in the fed state. We found that food ingestion was not significantly altered in the flies with DN-CREB expression in neurons (Figure 4A), indicating that reduced energy stores in the flies with DN-CREB expression is not due to reduced food intake. In contrast, despite their elevated lipid stores, food ingestion was increased in flies with DN-CREB expression in the fat body (Figure 4B). This result suggests that DN-CREB expression in the fat body disrupts detection of, or response to, the level of energy stores in the body.

### Adipokinetic hormone receptor in the fat body regulates CRE-mediated transcription in flies

In mammals, the pancreatic hormone glucagon activates CREB-mediated transcription in the liver [1]. The adipokinetic hormone (AKH) signaling pathway in *Drosophila* is functionally related to mammalian glucagon signaling [19], [20], [21], [22], [33], [34]. The receptor of AKH (AKHR) is expressed predominantly in the fat body and a subset of gustatory neurons [33], [34].

**Figure 5:**
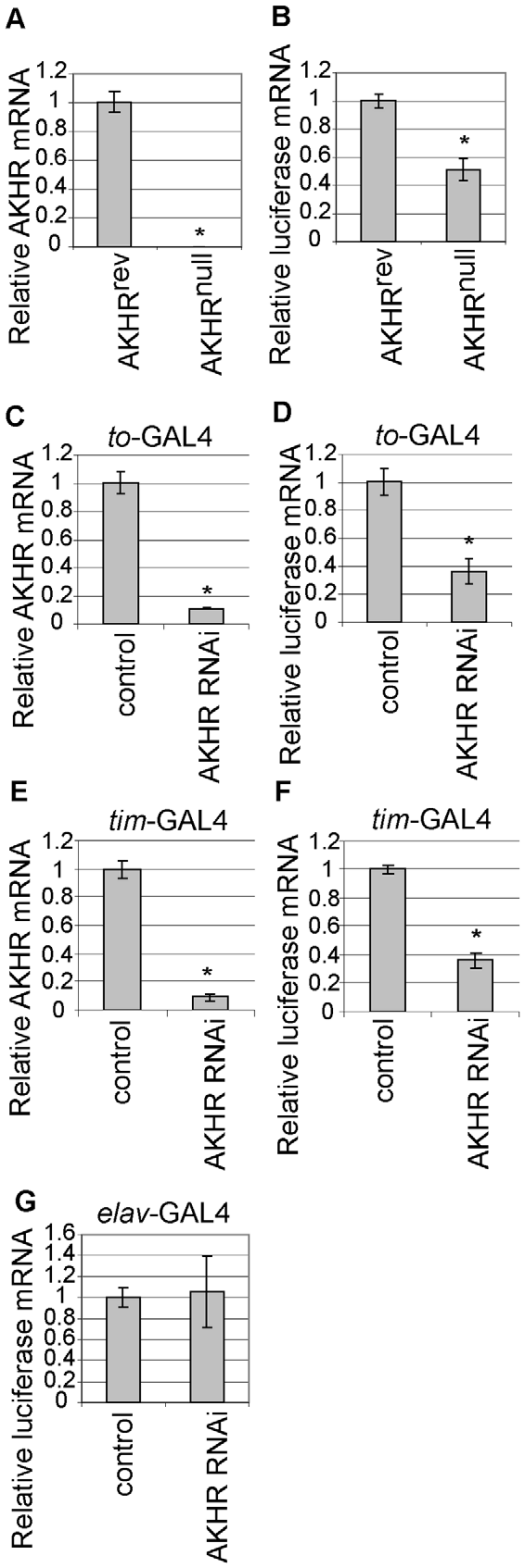
Reduction in CRE-mediated transcription by knock-down of AKHR in the fat body. Total RNA was extracted from flies and subjected to qRT-PCR. (A, C, and E) AKHR mRNA level, (B, D, F and G) CRE-luciferase mRNA levels. (mean±SD, n = 5, *p<0.05, Student's t-test).

To test whether AKHR plays a role in the regulation of CREB activity, we examined whether CRE-mediated transcription is altered in AKHR mutant flies using the CRE-Luciferase reporter transgene. Luciferase reporter mRNA levels were 50% lower in the AKHR mutant background than in the revertant control (AKHRrev) (Figure 5A and B). To determine whether AKHR in the fat body regulates CRE-mediated transcription, we knocked down AKHR expression in that tissue. Expression of AKHR RNAi driven by *to*-GAL4 caused a dramatic reduction in AKHR mRNA levels (Figure 5C) and a significant reduction in CRE-Luciferase reporter mRNA levels (Figure 5D). Similar results were obtained in flies expressing AKHR RNAi under the control of the *timeless*-GAL4 driver, which drives transgene expression strongly in the fat body [35] (Figure 5E and F). In contrast, expression of AKHR RNAi in neurons did not affect CRE-Luciferase reporter expression (Figure 5G). These results indicate that AKHR in the fat body positively regulates CRE-mediated transcription, and that the key signaling mechanism regulating CREB activity in peripheral tissues is evolutionarily conserved.

## Discussion

This study provides *in vivo* evidence that both neuronal and peripheral CREB activities are involved in the regulation of energy balance in flies. Blocking CREB activity in neurons caused reductions in both glycogen and lipid stores and a higher sensitivity to starvation stress. In contrast, while disruption of CREB function in the fat body also reduced glycogen levels, it increased lipid stores, and did not affect starvation sensitivity (Figure 2–3). Since there was no significant change in overall body size in these flies, disruption of CREB activity in the fat body caused an obese-like phenotype. These results also indicate that CREB activity can both increase and reduce lipid stores in flies depending on its site of action. Recently, two distinct populations of *Drosophila* brain neurons that regulate fat deposition were identified in *Drosophila* [36]. It will be interesting to determine in which neurons CREB functions to regulate energy metabolism in flies.

In a recent study, TORC-mediated CREB activity in neurons was shown to positively regulate glycogen and lipid stores in flies [4]. This is based on results showing that expression of TORC in neurons rescued the starvation sensitivity of TORC mutant flies. In addition, expression of TORC in neurons partially rescued the lower energy stores of these mutants [4]. While supporting the conclusions of this study with respect to the role of neuronal CREB activity, our results also provide evidence that CREB in the fat body plays roles in energy balance. Moreover, in contrast to the normal feeding behavior of a TORC mutant [4], we found that blocking CREB activity in the fat body increased food intake (Figure 4). Thus, disruption of CREB functions has a broader impact on energy metabolism and feeding behavior than the loss of TORC. It is likely that not all CREB functions depend on TORC. In support of this, although a TORC null mutant is viable and fertile [4], CREB mutants are lethal [23].

We found that AKH/AKHR signaling in the fat body, which is thought to be functionally related to glucagon/glucogon receptor signaling in the mammalian liver, positively regulates CRE-mediated transcription (Figure 5). In the mammalian liver, CREB activates the gluconeogenic program following a glucagon stimulus. Recent studies reported that promoting AKH signaling in the fat body significantly reduced, while loss of AKHR function modestly increased, glycogen levels in flies, presumably through AKH/AKHR-mediated carbohydrate catabolism in the fat body [33], [34]. However, we found that blocking CREB activity in the fat body significantly reduced glycogen levels (Figure 2), which would seem to contradict the proposed role of AKH/AKHR in mediating carbohydrate catabolism in the fat body. One possibility is that CREB activity in the fat body regulates multiple aspects of glucose/glycogen metabolism in addition to the AKH/AKHR-mediated pathway, and that blocking all CREB functions in the fat body reduces total glycogen levels as a net effect. In fact, significant CREB activity was remaining in AKHR mutant flies (Figure 5A), suggesting that other signaling pathways might contribute to the activation of CREB activity in the fat body. Further studies will be required to delineate the role of CREB activity in the fat body in carbohydrate metabolism and its relationship with the AKH signaling pathway.

We found that blocking CREB activity in the fat body increased lipid stores (Figure 2). AKH/AKHR is also thought to be functionally related to β-adrenergic signaling in mammalian adipose tissue, which activates protein kinase A (PKA) and stimulates lipolysis by phosphorylating hormone-sensitive lipase and perilipin [37], [38], [39]. In *Drosophila*, the promotion of AKH signaling in the fat body reduces lipid levels, whereas loss of AKHR function has the opposite effect; this is partly ascribed to altered activity in lipocatabolic systems [33], [34]. In addition, AKH signaling has been shown to repress the lipogenesis pathway in various insects [40], [41]. Interestingly, blocking CREB activity in mammalian liver causes excessive fat accumulation, resulting in “fatty liver” through overactivation of liposynthesis [2]. Future analysis will unravel whether CREB activity in the fat body represses liposynthesis and/or promotes lipid catabolism under the control of AKH/AKHR signaling.

In summary, our results demonstrate that CREB is involved in both central and peripheral regulation of energy balance and feeding behavior in *Drosophila*. Future studies of CREB in flies hold great promise for revealing the mechanisms underlying energy balance and feeding behavior. Such studies will likely contribute to our understanding of human metabolic disorders.

## Materials and Methods

### Fly stocks and culture

*dCREB2* ^S162^, hs-dCREB2d, UAS-dCREB2b, CRE-luciferase reporter (CRE-Luc) lines were described previously [23], [42], [43], [44]. Transgenic fly lines carrying hs-dCREB2d, UAS-dCREB2b, CRE-luciferase flies were established in the background of the Canton-S *w^1118^* (*isoCJ1*) genotype [25]. *takeout (to)*-GAL4 fly stock was a kind gifts from Dr. Amita Sehgal. *AKHR*null and *AKHR*rev flies were kind gifts from Dr. Kamal N. Bharucha [34]. UAS-AKHR RNAi (v9546) was obtained from the VDRC stock center (Vienna, Austria) [45]. The *elav-*GAL4^c155^ and *timeless*-GAL4 flies were obtained from the Bloomington *Drosophila* Stock Center (Indiana University). takeout is a member of a large family of secreted factors that bind small lipophiles, which was identified previously in several molecular screens as a robust circadian-regulated gene [46], [47], [48], [49] and plays a role in integrating information about the organism's sex, nutritional status, and circadian cycle to affect adult male behavior [31], [46], [50]. *timeless* is a circadian clock gene [51]. Expression pattern of the *to*-GAL4 and *timeless*-GAL4 drivers used in this study have been published (see references above). To obtain control flies for Figure 1, *isoCJ1* males were crossed to *yw* females, and F1 flies were subjected to the same heat-shock treatment and used as controls. To obtain control flies in for Figure 2–5, *isoCJ1* flies (Figure 2–4) or *w^1118^* from VDRC (Figure 5C–G) were crossed to the GAL4 drivers and F1 flies were used as controls. The flies were raised on standard cornmeal medium with 12 h:12 h light:dark cycle at 25°C.

### Western Blotting

Ten male flies were homogenized in Tris-glycine sample buffer (Invitrogen) and centrifuged at 13,000 rpm for 10 min, and the supernatants were separated on 10% Tris-glycine gels (Invitrogen) and transferred to nitrocellulose membranes (Invitrogen). The membranes were blocked with 5% nonfat dry milk (Nestlé) and blotted with the anti-dCREB2 antibody (a kind gift from Dr. J. Yin) or anti-luciferase antibody (Novus Biolocicals, Luci 21 1-107), incubated with appropriate secondary antibody and developed using ECL plus Western Blotting Detection Reagents (GE Healthcare). The signal intensity was quantified using ImageJ (NIH). Western blots were repeated a minimum of three times and representative blots are shown.

### Glycogen measurement

Glycogen levels were determined as described in [52]. 15–20 flies were quick frozen in liquid nitrogen, homogenized in 200 µl PBS containing 0.5% Tween 20, and immediately incubated at 70°C for 5 min. Samples were centrifuged and the supernatant was collected. Aliquot was incubated with 100 µl glucose reagent (Sigma) to measure the glucose level, or 100 µl glucose reagent and 0.3 U amyloglucosidase (Sigma) to measure the glycogen level. The samples were incubated at 37°C for 30 min and the absorbance at 540 nm was measured. Glucose and glucose plus glycogen amounts were determined using a standard curve and were normalized to the amount of protein. The amount of glycogen was determined by subtracting the amount of glucose from the glucose plus glycogen level. Experiments were repeated a minimum of three times.

### Triglyceride measurement

Triglyceride levels were determined as described in [52]. 15–20 flies were homogenized in 200 µl PBS, 0.5% Tween 20, and immediately incubated at 70°C for 5 min. Heat-treated homogenate was centrifuged, and supernatant were incubated with Triglyceride Reagent (Sigma) for 30 min at 37°C. Samples were then incubated with Free Glycerol Reagent (Sigma) for 5 min at 37°C, and assayed using spectrophotometer at 540 nm. Triglyceride levels were normalized to protein amounts in each homogenate, and data were analyzed using a Student's t test. Experiments were repeated a minimum of three times.

### Starvation assay

Male flies were kept in regular cornmeal food vials for 7 days after eclosion and then transferred to vials containing 1% agar. The number of dead flies were assessed every 6–12 h. About 50 flies were analyzed for each genotype. The assay was repeated at least three times and the representative data was shown.

### Oxidative stress assay

Male flies were kept in regular cornmeal food vials for 7 days after eclosion and then transferred to vials containing 20 mM paraquat/10% sucrose/PBS. The number of dead flies were assessed every 4–8 h. About 50 flies were analyzed for each assay. The assay was repeated at least three times and the representative data was shown.

### Food intake assay

Food intake assay was modified from [53]. 7–10 day-old males were placed in vials containing 1% FD&C Blue No. 1[MacCormick]/10% sucrose/1% agar for 6 h or 24 h. After the feeding, flies were frozen and bodies were homogenized in PBS and centrifuged twice for 25 min. The supernatant were transferred to cuvettes and absorbance was measured at 625 nm. Each experiment consisted of one or two groups of flies (20 flies each). The assay was repeated at least three times and the representative data are shown.

### Quantitative real-time PCR (qRT-PCR)

Total RNA was extracted from fly heads using TRIZOL reagent (Invitrogen). After reverse transcription using TaqMan Reverse Transcription Reagents (Applied Biosystems), qRT-PCR reactions were performed with SYBR PCR Master Mix Reagents and analyzed with a Sequence Detection System 7700 (Applied Biosystems). Relative expression values were determined by the ΔΔ*Ct* method according to quantitative PCR Analysis User Bulletin (Applied Biosystems). Primers were designed using Primer Designing Tool (NCBI): Luciferase, TTGGATCTT CCAGGGATACGA (forward) and TTTCCCGGTATCCAGATCCA (reverse), AKHR, TCCATCACCGTGTACAGCAT (forward) and GAGCGATATGCAGACCATCA (reverse), Actin, TGCACCGCAAGTGCTTCTAA (forward) and TGCTGCACTCCAAACTTC CA (reverse).
